# Decreased plasma soluble erythropoietin receptor in high-altitude excessive erythrocytosis and Chronic Mountain Sickness

**DOI:** 10.1152/japplphysiol.00619.2014

**Published:** 2014-10-16

**Authors:** Francisco C. Villafuerte, José Luis Macarlupú, Cecilia Anza-Ramírez, Daniela Corrales-Melgar, Gustavo Vizcardo-Galindo, Noemí Corante, Fabiola León-Velarde

**Affiliations:** Laboratorio de Fisiología Comparada, Departamento de Ciencias Biológicas y Fisiológicas, Facultad de Ciencias y Filosofía, Universidad Peruana Cayetano Heredia, Lima, Peru

**Keywords:** Chronic Mountain Sickness, excessive erythrocytosis, erythropoietin, soluble erythropoietin receptor, high-altitude

## Abstract

Excessive erythrocytosis (EE) is the hallmark of chronic mountain sickness (CMS), a prevalent syndrome in high-altitude Andean populations. Although hypoxemia represents its underlying stimulus, why some individuals develop EE despite having altitude-normal blood erythropoietin (Epo) concentration is still unclear. A soluble form of the Epo receptor (sEpoR) has been identified in human blood and competes directly for Epo with its membrane counterpart (mEpoR). Thus, reduced levels of circulating sEpoR could lead to higher Epo availability and ultimately to EE. We characterized the relationship between Epo and sEpoR, with hematocrit and hemoglobin concentration in healthy highlanders and CMS patients at 4,340 m in Cerro de Pasco, Peru. Our results show that EE patients show decreased plasma sEpoR levels and can be subdivided into two subgroups of normal and high plasma Epo concentration for the altitude of residence, with hemoglobin concentration rising exponentially with an increasing Epo-to-sEpoR ratio (Epo/sEpoR). Also, we showed that the latter varies as an inverse exponential function of arterial pulse O_2_ saturation. Our findings suggests that EE is strongly associated with higher Epo/sEpoR values, leading to elevated plasma Epo availability to bind mEpoR, and thereby a stronger stimulus for augmented erythropoiesis. Differences in the altitude normal and high Epo CMS patients with a progressively higher Epo/sEpoR supports the hypothesis of the existence of two genetically different subgroups suffering from EE and possibly different degrees of adaptation to chronic high-altitude hypoxia.

excessive erythrocytosis (EE; Hb concentration ≥ 21 g/dl) is the main sign of chronic mountain sickness (CMS), a clinical syndrome associated with severe hypoxemia and neurological symptoms, such as headache, fatigue, somnolence, and alterations of sleep and memory (27, 36, 38). Pulmonary hypertension is a frequent complication of the condition and can lead to right-heart failure in the end stages of the disease (39, 40).

CMS normally affects people who reside at altitudes >2,500 m above sea level (28, 37), and it is estimated that on average, 5–10% of the world's population living at high-altitude may develop EE and CMS (28). Epidemiological studies in the central Andes of Peru >4,000 m have shown that CMS occurs in 16% of the adult male population, and that its prevalence increases with age, rising up to 30% by the fifth decade of age (27, 35).

The pathophysiological mechanism of EE and CMS is still debatable; however, hypoxemia represents the underlying stimulus as relocation of CMS patients to sea level completely reverts the disorder. Also, studies at high altitude have shown that phlebotomy, with or without isovolemic hemodilution, alleviates the CMS-related symptoms, suggesting that many of these are associated with EE.

Erythropoiesis is primarily regulated by erythropoietin (Epo), the synthesis of which depends on arterial Po_2_ and therefore on arterial O_2_ saturation (SaO_2_). Thus hypoxemia represents the main stimulus for the erythropoietic process (19, 21, 22). Studies in healthy highlanders have shown higher circulating Epo concentration compared with sea level values and, accordingly, a higher Hct and Hb concentration (13, 15, 30). It would, therefore, be expected to find a higher Epo concentration in subjects with EE.

Decreased ventilation during daytime or during sleep has been proposed as a possible factor that contributes to arterial hypoxemia, which, in turn, may enhance Epo production and trigger EE (24, 25, 36, 37). Also, altered circadian patterns of Epo production (8) and other hormonal factors (15, 31) have been suggested to contribute to this condition. However, there is controversy whether CMS patients have elevated circulating Epo concentration, as some studies have found similar serum Epo values in these individuals compared with healthy highlanders (15, 30), whereas others have not (41). These findings reflect great variability in blood Epo values at altitude and support the idea of two subgroups of highlanders with EE: one with normal Epo concentration for the altitude of residence, and the other with significantly higher Epo values (12, 30). Moreover, we have shown that there is a poor correlation between arterial pulse O_2_ saturation (Sp_O_2__) and blood Epo levels in Andean healthy highlanders (30). Therefore, why some individuals develop EE, despite having similar Sa_O_2__ and/or blood Epo concentration, is still unclear.

Epo exerts its action on erythropoiesis by promoting the survival, proliferation, and differentiation of erythrocytic progenitors. By binding its membrane receptor (mEpoR), Epo acts as an anti-apoptotic agent for these cells, predominantly the colony-forming units-erythroid (CFU-E). In response to Epo, CFU-Es proliferate and differentiate to generate cohorts of proerythroblasts and normoblasts, which then mature into reticulocytes in the bone marrow. Once in the bloodstream, reticulocytes mature into erythrocytes (22, 34). Therefore, the hypoxia-induced increase in circulating Epo accelerates this process, increasing the rate of red blood cell production to increase blood O_2_-carrying capacity (19).

It has been suggested that mEpoR might not be the only EpoR involved in the physiological regulation of erythopoiesis, as a soluble form of EpoR (sEpoR) has been identified and isolated from human blood (5, 17, 44, 46). The sEpoR corresponds to the extracellular domain of the complete receptor (17, 38, 46), and its synthesis occurs by alternative splicing of EpoR mRNA (14, 18, 46). Following its secretion into the extracellular fluid, sEpoR binds Epo, thereby limiting its ability to bind mEpoR (5, 32, 43). Thus sEpoR acts as an Epo “buffer,” regulating available circulating Epo concentration. A lower sEpoR concentration, for example, would increase unbound free plasma Epo and, therefore, its availability for binding mEpoR.

Several studies have provided evidence for a role of sEpoR in modulating Epo signaling as sEpoR blocks Epo signaling in vitro and in vivo by direct competition for Epo with mEpoR (5, 26, 32, 43). The presence of sEpoR has been reported in plasma and several tissues, including liver, spleen, kidney, muscle, brain, and bone marrow (14, 32, 43).

A recent study has suggested that sEpoR plays a role in the physiological regulation of erythropoiesis by decreasing the available Epo concentration in blood and, consequently, decreasing erythropoietic activity (23). In addition, it has been demonstrated that chronic hypoxia decreases the expression of sEpoR (43). Accordingly, we hypothesized that a reduction in circulating sEpoR in CMS leads to a higher Epo availability and, therefore, to the development of EE. This possible modulation of Epo availability by sEpoR could also explain why some high-altitude dwellers develop EE, despite having similar SaO_2_ and circulating Epo concentration.

The aim of the present study was to characterize the relationship between plasma Epo and sEpoR and Hb concentration in Andean healthy highlanders and CMS patients.

## METHODS

This study was approved by the Institutional Ethics Committee of Universidad Peruana Cayetano Heredia (Lima, Peru). Participants provided written, informed consent.

### 

#### Study participants.

Eighty-six highlander participants (CMS, *n* = 42; healthy highlanders, *n* = 44) in the age range from 20 to 65 yr, from the city of Cerro de Pasco, Peru, at 4,340 m, were recruited for the study. Also, healthy participants from sea level (*n* = 25) of the same age range were recruited for comparison. Table 1 summarizes the characteristics of these groups. Exclusion criteria were the presence of pulmonary, cardiovascular, or renal disease, recent phlebotomy (<1 yr), and travel to lower altitude during the previous year. Participants underwent clinical examination, which included Sp_O_2__ measurement as an indicator of Sa_O_2__. Participants also answered a general health and a CMS Score questionnaire (28).

**Table 1.** Characteristics of study participants

	Healthy Highlanders	CMS Patients	Sea Level Residents
Age, yr	39.2 ± 1.85	44.2 ± 1.87	33.8 ± 2.13
BMI, kg/m^2^	24.6 ± 0.45	25.8 ± 0.49	26.2 ± 0.80
Hematocrit, %	52.9 ± 0.44	68.0 ± 0.76‡	43.4 ± 0.51‡
CMS score	2.7 ± 0.38	7.4 ± 0.58‡	1.4 ± 0.30†
Arterial O_2_ saturation, %	88.2 ± 0.62	84.0 ± 0.59‡	98.3 ± 0.20‡
Iron, μg/dl	110.3 ± 7.82	124.6 ± 13.50	87.3 ± 4.81
Ferritin, ng/ml	136.2 ± 14.27	183.2 ± 22.44	158.7 ± 18.12
Transferrin saturation, %	32.8 ± 2.38	32.8 ± 3.08	25.4 ± 1.38*

Values are means ± SE; healthy highlanders (*n* = 44), chronic mountain sickness (CMS) patients (*n* = 42), sea level residents (*n* = 25). BMI, body mass index. Significance vs. healthy highlanders:

* *P* < 0.05,

† *P* < 0.01,

‡ *P* < 0.001.

Previous studies have suggested that highlanders with CMS can be classified into two subgroups according to their plasma Epo concentration (12, 30). Therefore, in the present study, the CMS group was subdivided into those with values similar to healthy highlanders (normal-Epo CMS), and those with significantly higher plasma Epo concentration (high-Epo CMS). A cutoff value of 2 SD above the mean plasma Epo concentration of healthy highlanders was chosen and employed to define the high-Epo CMS group.

#### Samples.

Blood samples were taken between 7 AM and 10 AM to avoid variation in plasma Epo due to circadian rhythm. Plasma and serum were separated immediately and stored at −20°C, and then in liquid N_2_ until analysis. Hct and Hb concentration were determined by microcentrifugation and a Hemocue HB201 system (HemoCue AB), respectively.

#### Hematological indexes of iron homeostasis.

To assess the iron status of participants; serum iron, transferrin, and ferritin were quantified. The stability of these markers in frozen samples has been reported previously (20). Serum iron and transferrin concentration were measured photometrically (multianalyzer CB350 i, Wiener Laboratory), and ferritin concentration was measured by chemiluminescence (multianalyzer Cobas e 411, Roche Diagnostics) at the clinical laboratory facilities of the Cayetano Heredia Clinic (ISO 9007–2008), Lima, Peru.

#### Epo and sEpoR determination.

Total plasma Epo and sEpoR concentrations were quantified using specific sandwich ELISA kits (USCN Life Science, Houston, TX). The standard sample storage and analysis procedure described by the manufacturer was followed for each kit. Briefly, 100 μl of plasma were added to each well, which had been precoated with a mouse-raised monoclonal capture antibody specific to human Epo or EpoR and incubated in a dry bath at 37°C for 2 h. Immediately after, excess sample was discarded, and, without washing, a specific biotin-conjugated rabbit-derived polyclonal antibody was added and incubated for 1 h at 37°C. The plate was then washed three times, and avidin-conjugated horseradish peroxidase was added and incubated at 37°C for an additional 30 min. After this, the plate was washed five times, and a tetramethylbenzidine substrate solution was added and incubated for 15 min at 37°C. The reaction was stopped by the addition of sulfuric acid. Absorbance was immediately measured at 450 nm with a background wavelength of 630 nm. Samples were run in duplicate. Recombinant human Epo was used for the standard calibration curve at 400, 200, 100, 50, 25, 12.5, and 6.25 pg/ml, while recombinant human EpoR was used at concentrations of 10, 5, 2.5, 1.25, 0.625, 0.312, and 0.156 ng/ml. The standard diluent was used as blank for each assay. The minimum detectable concentration of Epo and sEpoR for these assays is typically less than 2.36 pg/ml and 0.057 ng/ml, respectively. Antibodies for the receptor provided in the kit have been previously tested for specific recognition by immunohistochemical staining and Western blot and measure total receptor concentration by binding to both the free and bound forms. Detection of sEpoR in the assay was verified by using lyophilized soluble receptor isolated from human plasma at a concentration of 0.79 ng/ml as a positive control provided by the manufacturer.

#### Statistical data analysis.

STATA 11 software was employed for statistical analysis. For comparison of means between groups, the normality of distribution and homogeneity of variance of all continuous variables were assessed to determine the use of parametric or nonparametric tests. Student's *t*-tests for equal and unequal variances were applied as parametric tests of comparison, and Wilcoxon as nonparametric, to evaluate differences between healthy highlanders and CMS patients, and to confirm differences between healthy highlanders and sea level individuals reported in previous studies. ANOVA and Kruskal-Wallis were applied as multiple-comparison tests between healthy highlanders, normal-Epo CMS, and high-Epo CMS groups as parametric and nonparametric tests, respectively. Differences were considered significant if *P* < 0.05 with a statistical power >0.85. Additionally, two stepwise regressions were performed to determine the independent variables among the possibly associated parameters [Sp_O_2__, age, body mass index (BMI), Epo, sEpoR, Epo-to-sEpoR ratio (Epo/sEpoR), iron, ferritin, and transferrin] that show significant influence (*P*_*e*_ = 0.05 and *P*_*r*_ = 0.1) over the dependent variable (Hb). Since Epo, sEpoR, and Epo/sEpoR present multicollinearity when included in the same model, one stepwise regression included the parameters separately and the other the ratio, along with the other independent variables mentioned above. The resultant multiple regression models were evaluated for normality and homoscedasticity. Only the models that fulfilled these requirements were incorporated in the study.

## RESULTS

No differences in age or BMI were observed between groups. Hct and CMS score were significantly higher in the CMS group compared with healthy highlanders, whereas mean Sp_O_2__ was significantly lower, as has been previously reported. Iron homeostasis parameters were similar between the altitude groups, but, as expected, differed in serum iron concentration and transferrin saturation with respect to sea level values (Table 1).

The CMS group was divided into normal-Epo CMS (*n* = 32) and high-Epo CMS (*n* = 10) subgroups according to their plasma Epo values (see methods; Table 2).

**Table 2.** Characteristics of normal-Epo and high-Epo CMS subgroups

	Normal-Epo CMS	High-Epo CMS
Age, yr	44.5 ± 2.42	43.6 ± 4.38
BMI, kg/m^2^	25.7 ± 0.60	26.6 ± 1.08
Hematocrit, %	66.6 ± 0.53	75.9 ± 2.65*
CMS score	7.2 ± 0.87	8.00 ± 1.38
Arterial O_2_ saturation, %	84.4 ± 0.72	82.5 ± 1.05
Iron, μg/dl	128.7 ± 16.52	118.2 ± 23.93
Ferritin, ng/ml	183.9 ± 27.55	187.1 ± 44.86
Transferrin saturation, %	33.2 ± 3.68	31.1 ± 7.38

Values are means ± SE; normal-erythropoietin (Epo) CMS (*n* = 32), high-Epo CMS (*n* = 10). Significance versus normal-Epo CMS subgroup:

* *P* < 0.001.

**Fig. 1.**
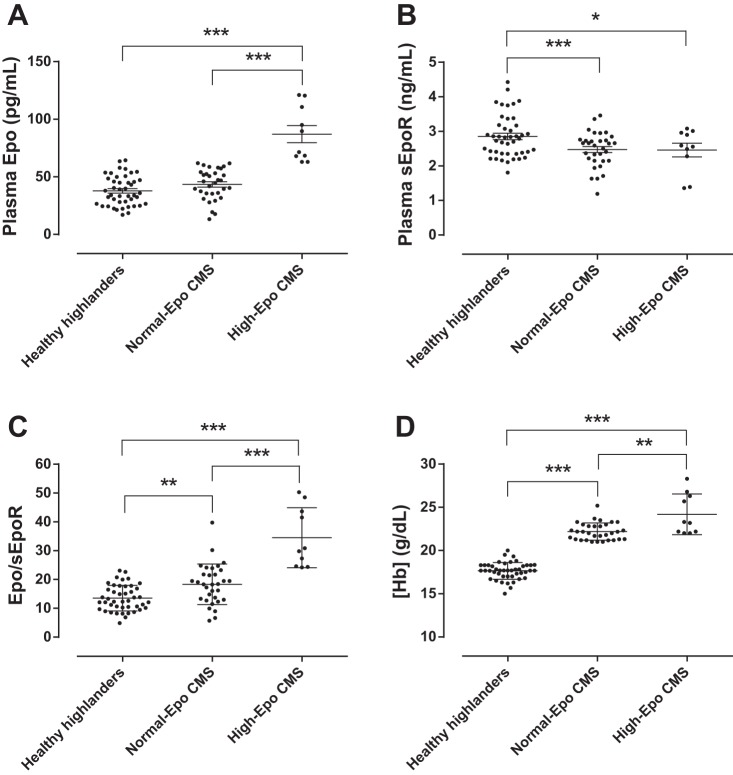
Erythropoiesis-related parameters in healthy highlanders and Chronic Mountain Sickness (CMS) patients. The figure shows the scatter graphs with individual values in each group for Hb concentration ([Hb]; *A*), plasma erythropoietin (Epo) concentration (*B*), plasma soluble Epo receptor (sEpoR) concentration (*C*), and the Epo-to-sEpoR ratio (Epo/sEpoR; *D*) between healthy highlanders and CMS individuals with normal and high plasma Epo concentration (Normal-Epo CMS and High-Epo CMS, respectively) at high-altitude. Horizontal lines represent means ± SE. *P < 0.05, **P < 0.01, ***P < 0.001.

**Fig. 2.**
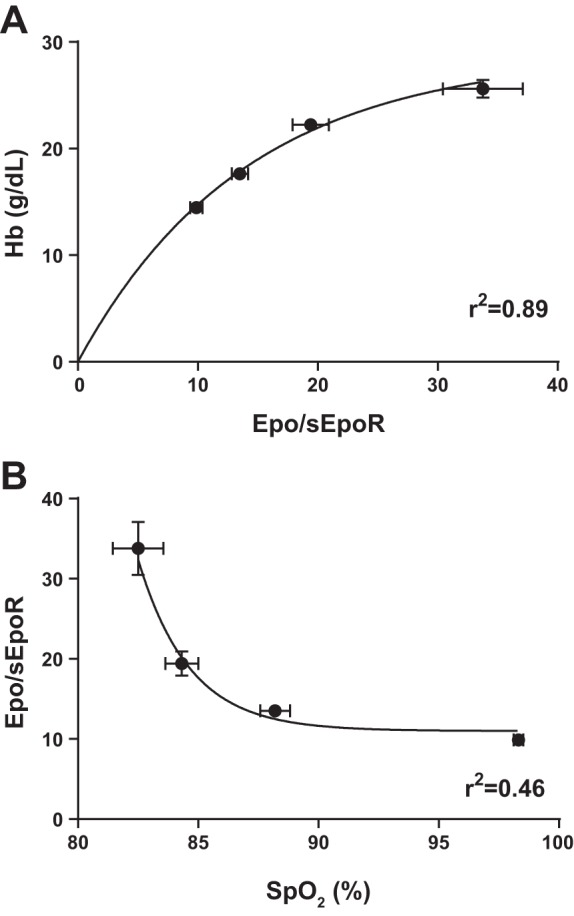
Relationship between the Epo/sEpoR, [Hb], and arterial pulse O_2_ saturation (Sp_O_2__). The figure shows nonlinear best fit for mean Hb values as a function of mean Epo/sEpoR (*A*), and for mean Epo/sEpoR values as a function of Sp_O_2__ (*B*), including sea level residents, healthy highlanders, and Normal-Epo CMS and High-Epo CMS patients. Values are means ± SE.

Plasma Epo concentration was higher in the high-Epo CMS group than in both normal-Epo CMS and healthy highlander groups (87.1 vs. 43.5 and 37.8 pg/ml, *P* < 0.05), while no significant difference was found between these last two (Fig. 1*A*). sEpoR concentration was 16% lower in the normal-Epo CMS and high-Epo CMS groups compared with the healthy group (2.48 and 2.46 vs. 2.86 ng/ml, *P* < 0.05; Fig. 1*B*). sEpoR concentration values were within the range published in previous studies (9, 46). To obtain an index of plasma Epo availability, Epo/sEpoR was calculated. Epo/sEpoR was highest in the high-Epo CMS and normal-Epo CMS groups, followed by the healthy group (*P* < 0.001, Fig. 1*C*). The high-Epo CMS group showed the highest Hb concentration, followed by normal-Epo CMS patients and healthy highlanders (24.2, 22.2, and 17.7 g/dl, respectively, *P* < 0.01; Fig. 1*D*). Also, Hb concentration varied exponentially with an increasing Epo/sEpoR from sea level to high-Epo CMS values (Fig. 2*A*), and the latter varied as an inverse exponential function of Sp_O_2__ (Fig. 2*B*).

### 

#### Multivariate model.

Stepwise regression analysis showed that the independent variables in the multiple regression model (which included Sp_O_2__, age, BMI, Epo, sEpoR, iron, ferritin, and transferrin) with significant influence over Hb were Sp_O_2__, Epo, and sEpoR (model I, Table 3). Similarly, in the model including the Epo/sEpoR instead of the separate parameters, the stepwise analysis showed significant influence from Sp_O_2__ and the ratio (model II, Table 3). The sEpoR had the highest regression coefficient, followed by Sp_O_2__, while Epo showed little influence.

**Table 3.** Multiple regression models between Hb concentration and Sp_O_2__, Epo, sEpoR, and Epo/sEpoR

			Parameters				
Dependent Variable	Model	Independent Variables	Coefficient of Regression (β)	SE	*P* Value	Confidence Interval (95%)	
Hb concentration	I	Sp_O_2__	−0.25	0.054	<0.001	−0.356	−0.142
		Epo	0.07	0.012	<0.001	0.041	0.090
		sEpoR	−0.84	0.403	<0.001	−1.641	−0.039
		Constant	40.87	4.717	<0.001	31.48	50.25
	II	Sp_O_2__	−0.26	0.054	<0.001	−0.368	−0.151
		Epo/sEpoR	0.14	0.028	<0.001	0.085	0.198
		Constant	39.95	4.844	<0.001	30.31	49.58

Sp_O_2__, arterial pulse O_2_ saturation; sEpoR, soluble form of the Epo receptor; Epo/sEpoR, ratio of Epo to sEpoR.

## DISCUSSION

At altitude, healthy highlanders maintain higher Hct and Hb values with modestly increased blood Epo concentration compared with their lowland counterparts (13, 15, 30). This observation follows a straightforward rationale, given that Hct and Hb concentration depend on blood Epo, and the latter depends on Sa_O_2__ (16). Accordingly, a higher circulating Epo concentration would be expected in highlanders with low Sa_O_2__ and EE. It has been reported, however, that Epo concentration is mostly similar between healthy and CMS highlanders at the same altitude (15, 30), and that there is no correlation between serum Epo levels and Hb concentration, revealing a large phenotypic variation. It seems paradoxical then that, with similar circulating Epo, some highlanders develop EE and others do not.

Quantitative genetic analysis revealed that the proportion of phenotypic variance in Hb concentration attributable to genetic factors is ∼87% in Aymaran Andeans of La Paz, Bolivia (6). Also, it has been shown that high Hb values in Quechua Andeans have a familial component (29). These studies point out a genetic factor for EE, although no specific genetic factors underlying intrapopulation Hb concentration variance have been identified in Quechua population.

Recent studies have proposed some genetic characteristics associated with Hb concentration in Tibetan high-altitude natives. It has been suggested that these characteristics confer protection to this population against the development of EE (7, 42, 48). Specific single-nucleotide polymorphisms (SNPs) in the *EPAS1* and *EGLN1* genes have been shown to be associated with low Hb concentration values in Tibetans. The products of these genes, HIF-2α and prolyl hydroxylase domain enzyme 2 (PHD2), respectively, are major regulators of erythropoiesis, given their central role in the control of the hypoxic response of Epo expression at the transcriptional level. Therefore, it is possible that differences in these genes, and also in the genes encoding for Epo and EpoR, might explain why only some individuals of the same population at a given altitude develop EE, such as in the case of Andeans.

So far, the few genetic studies using candidate-gene approach have not been able to show clear evidence of the involvement of genes of the HIF pathway, or the Epo or EpoR genes in the development of EE and CMS in Andeans (2, 33, 29). Although some differences have been reported at the gene expression level (2, 3, 29), such as enhanced expression of HIF-1α and VEGF in peripheral blood leukocytes obtained from CMS patients, these differences fail to explain the occurrence of EE within the same population, and in some cases (i.e., VEGF expression), appear to be more of a consequence of CMS rather than a cause.

The fact that EE develops with circulating Epo concentrations similar to those of healthy highlanders raises the possibility that sensitivity to Epo or in the Epo system is increased in CMS patients. An in vitro preliminary study has suggested that the earliest erythroid progenitors, the burst-forming unit-erythroid (BFU-E), isolated from CMS patients are more sensitive to recombinant human Epo and have prolonged viability compared with those of healthy high-altitude dwellers (1). The mechanism by which this occurs is unknown. Whether it lies on differences in the intracellular Epo signaling cascade or on a higher hypoxia-induced density of mEpoR is still uncertain. It is possible that increased sensitivity of these progenitors represents the cellular level component that leads to EE.

However, it has been suggested that the modulation of the sensitivity of the erythropoietic response to Epo also has an extracellular component.

Several studies have provided evidence for a physiological role of sEpoR by direct competition for Epo with mEpoR, and also for an effect of hypoxia on sEpoR levels (5, 9, 23, 26, 32, 43). It has been shown, for example, that changes in plasma sEpoR concentration influence the effect of Epo on Hb concentration (23). Also, 3-day normobaric hypoxia exposure can downregulate sEpoR expression in mice brain by 62% (43). Additionally, intermittent 8-day hypoxia exposure (6 h/day, 2-min hypoxia followed by 2-min reoxygenation; peak end-tidal and minimal Po_2_, 88 and 45 Torr, respectively) downregulates plasma sEpoR by 70% on the second day with a concomitant peak rise in Epo concentration of 50% in humans (9). Plasma sEpoR then increases as Epo falls until both reach a new steady state. Despite the short duration of hypoxic exposure used in these studies, the evidence of a dynamic interplay between Epo and sEpoR suggests that the latter might be part of a fine-tuned mechanism that regulates Epo availability and, therefore, the erythropoietic stimulus. This mechanism is similar to that reported for other soluble cytokine and growth factor receptors generated by alternative mRNA splicing (18). The effects of interleukin-4, for example, are positively regulated by its membrane-bound receptor and negatively by the soluble receptor form (11). Another example includes the soluble epidermal growth factor receptor, which acts as an antagonist and competes with the membrane receptor for binding its ligand epidermal growth factor (5).

The fact that sEpoR levels show a decrease with hypoxic exposure suggests that EpoR mRNA splicing machinery is hypoxia sensitive. Changes on alternative splicing of several genes have been reported as a physiological response to hypoxia, whether by upregulating or downregulating splice isoform expression (45). Thus it is possible that chronic hypoxemia is associated with long-term alteration of EpoR mRNA splicing, which leads to downregulation of the sEpoR isoform.

At present, there is no information on the effect of lifelong chronic hypoxia exposure on sEpoR. Our results demonstrate that highlanders with EE of the normal-Epo CMS group show lower Sp_O_2__ and sEpoR levels, but similar circulating Epo concentration compared with healthy highlanders, which results in a higher Epo/sEpoR and probably contributes to a stronger erythropoietic stimulus. The fact that the greater hypoxemia in this group is not associated with higher plasma Epo, but is associated with decreased sEpoR levels, suggests that chronic hypoxia affects Epo and sEpoR in a differential manner, and that probably some of the genetic component of EE and CMS is reflected in a chronic downregulation of the sEpoR production.

An interesting pattern emerges when the high-Epo CMS group is analyzed. This group also shows lower Sp_O_2__ and sEpoR levels compared with that in healthy highlanders, but, compared with the normal-Epo group, exhibit similar Sp_O_2__ and sEpoR, higher Epo concentration, and slightly higher Hb concentration. This observation suggests that there might be a difference within the CMS group in terms of Epo expression.

Recently, whole-genome sequencing in Andeans (4,340 m) identified large frequency differential SNPs between CMS and non-CMS individuals in *SENP1*, a candidate gene suggested to regulate erythropoiesis (49). The *SENP1* gene showed upregulated expression (2-fold) upon hypoxic challenge (1.5% O_2_ for 24 h) in cultured skin fibroblasts obtained from these CMS individuals compared with healthy highlanders. Interestingly, studies in mice during embryonic development have shown that *SENP1* triggers erythropoiesis via stabilization of the HIF-1α subunit and subsequent stimulation of Epo expression (10), and also by stabilizing GATA1, a transcription factor that drives the expression of many erythropoietic genes, including EpoR (47). However, it is not yet known whether these mechanisms also play a role in Epo and EpoR expression and erythropoiesis in adult humans.

This is the first study to report the pattern of Epo and sEpoR in healthy Andean highlanders and in highlanders with EE with normal and high plasma Epo concentration. The existence of three subgroups of highlanders (healthy, normal-Epo CMS, and high-Epo CMS) suggests a differential regulation of Epo and sEpoR, and a physiological role of sEpoR in the modulation of the erythropoietic stimulus. Moreover, the physiological differences in the Epo system among the groups and their erythropoietic response might indicate specific underlying genetic backgrounds, which might, in turn, reflect different levels of adaptation to lifelong high-altitude hypoxia. In other words, the high-Epo CMS group, with its exaggerated erythrocytosis and elevated plasma Epo, could be regarded as the least adapted, followed by the normal-Epo CMS group, with still excessive but lower Hb and normal Epo for the altitude of residence, and finally the healthy Andeans, with a modestly elevated Hb and normal Epo values, as the most adapted among the groups.

In conclusion, our results suggest that the severity of erythrocytosis in high-altitude dwellers depends on the interaction of circulating Epo and sEpoR, and not necessarily only on blood Epo concentration; and that EE is strongly associated with decreased sEpoR levels and with a higher Epo/sEpoR. CMS patients with normal and high Epo concentration show decreased plasma sEpoR compared with healthy highlanders, which, therefore, results in higher Epo/sEpoR, higher Epo availability, and thus higher Hb concentration. Therefore, the Epo/sEpoR can be considered an index of plasma Epo availability and ultimately of the intensity of erythropoietic stimulus. Decreased sEpoR levels in CMS patients compared with healthy highlanders probably reflects the effect of chronic excessive hypoxemia combined with underlying genetic differences within the same high-altitude population. Finally, our finding of normal and high Epo CMS patients with progressively higher Epo/sEpoR supports the hypothesis of the existence of two genetically different subgroups suffering from EE, and different degrees of adaptation to chronic high-altitude hypoxia.

## GRANTS

This research was supported by a Wellcome Trust Public Health and Tropical Medicine Fellowship (097275/Z/11/Z) to F. C. Villafuerte.

## DISCLOSURES

No conflicts of interest, financial or otherwise, are declared by the author(s).

## AUTHOR CONTRIBUTIONS

Author contributions: F.C.V. conception and design of research; F.C.V., J.L.M., C.A.-R., D.C.-M., and G.V.-G. performed experiments; F.C.V., C.A.-R., D.C.-M., N.C., and F.L.V. analyzed data; F.C.V., D.C.-M., N.C., and F.L.V. interpreted results of experiments; F.C.V. prepared figures; F.C.V. and N.C. drafted manuscript; F.C.V., C.A.-R., D.C.-M., N.C., and F.L.V. edited and revised manuscript; F.C.V., J.L.M., C.A.-R., D.C.-M., G.V.-G., N.C., and F.L.V. approved final version of manuscript.
